# ﻿The genus *Platydracus* Thomson in Hainan, South China (Coleoptera, Staphylinidae, Staphylininae)

**DOI:** 10.3897/zookeys.1212.128295

**Published:** 2024-09-16

**Authors:** Jia-Yi Huang, Jia-Wei Chen, Liang Tang

**Affiliations:** 1 College of Life Sciences, Shanghai Normal University, 100 Guilin Road, 1st Educational Building 323 Room, Shanghai, 200234, China Shanghai Normal University Shanghai China; 2 Diaoluoshan Branch, Hainan Tropical Rainforest National Park Administration, Lingshui County, Hainan, 572400, China Diaoluoshan Branch, Hainan Tropical Rainforest National Park Administration Hainan China

**Keywords:** identification key, new record, new species, *Platydracus*, rove beetle, taxonomy

## Abstract

A study on *Platydracus* species of Hainan Province, China is presented. *Platydracushainanensis***sp. nov.**, *Platydracusaureolus***sp. nov.** and *Platydracuszhouchenglini***sp. nov.** are described as new species. *Platydracusmarmorellus* Fauvel, 1895 and *Platydracussubirideus* Kraatz, 1859 are recorded from China for the first time. Habitus and diagnostic characters of all species are photographed, and a key to *Platydracus* species of Hainan is provided.

## ﻿Introduction

*Platydracus* Thomson, 1858 is the largest genus among the subtribe Staphylinina Latreille, 1802. Up to the present, around 300 species have been described in the genus and 32 of them are known from China. Hainan is a large island located in the South China with high insect diversity. Yet only one species, *Platydracusjuang* Smetana, 2005, has been presently recorded in this area (Zhou et al. 2024). After conducting several field investigations in Hainan, many staphylinid specimens were collected. Among them, six *Platydracus* species are recognized, including three new species which will be described in this paper.

## ﻿Materials and methods

The specimens examined in this paper were collected by searching dead wood, sifting leaf litter and setting flight interception traps (FIT). For examination of the genitalia, the last three abdominal segments were detached from the body after softening in hot water. The aedeagus or tergite X, together with other dissected pieces, were mounted in Euparal (Chroma Gesellschaft Schmidt, Koengen, Germany) on plastic slides. Photos of sexual characters were taken with a Canon G9 camera attached to an Olympus SZX 16 stereomicroscope; habitus photos were taken with a Canon MP-E 65 mm macro lens attached to a Canon EOS7D camera and stacked with Zerene Stacker.

The specimens treated in this study are deposited in the Department of Biology, Shanghai Normal University, P. R. China (SHNU), Natural History Museum of Denmark at the University of Copenhagen, Denmark (NHMD), the Canadian National Collection of Insects, Arachnids and Nematodes, Ontario, Canada (CNC) and the National Museum Prague, Czech Republic (NMPC).

### ﻿Body measurements are abbreviated as follows

**BL** body length, measured from anterior margin of clypeus to posterior margin of abdominal tergite X

**FL** forebody length, measured from anterior margin of clypeus to apex of elytra (apicolateral angle)

**HL** length of head along midline

**HW** width of head including eyes

**EYL** length of eye

**TL** length of tempora

**PL** length of pronotum along the midline

**PW** width of pronotum at the widest point

**EL** length of elytra, measured from humeral angle

**EW** width of elytra at the widest point

## ﻿List of *Platydracus* species of Hainan

*Platydracusaureolus* sp. nov.

*Platydracushainanensis* sp. nov.

*Platydracusjuang* Smetana, 2005

*Platydracusmarmorellus* Fauvel, 1895

*Platydracussubirideus* Kraatz, 1859

*Platydracuszhouchenglini* sp. nov.

### ﻿Key to *Platydracus* species of Hainan

**Table:** 

1	Pronotum with complete impunctate midline; male abdominal sternite VII with a large median depression bearing numerous long golden setae	**2**
–	Pronotum without complete impunctate midline; male abdominal sternite VII without large median depression bearing numerous long golden setae, at most bearing a small cluster of golden setae	**3**
2	Elytra with copper tint; head with punctation coarser and denser (Fig. 1)	***P* . *hainanensis* sp. nov.**
–	Elytra without copper tint; head with punctation finer and sparser (Fig. 2)	***P.juang***
3	Majority of head and pronotum covered with two types of setae: setae and appressed microsetae	**4**
–	Majority of head and pronotum only covered with one type of setae	**5**
4	Head bicolorous; abdominal tergite VIII yellowish, distinctly lighter than previous tergites	***P.marmorellus***
–	Head uniformly blackish; abdominal tergite VIII brownish, same color as previous tergites	***P.subirideus***
5	Larger species, BL = 21.9–25.2 mm; head and pronotum with distinct golden tint (Fig. 17); abdominal tergites VI–VII entirely covered with golden setae (Fig. 17)	***P* . *aureolus* sp. nov.**
–	Smaller species, BL = 9.5–16.8 mm; head and pronotum without metallic tint (Fig. 21); abdominal tergites VI–VII with golden setae only medially (Fig. 21)	***P.zhouchenglini* sp. nov.**

### ﻿Taxonomy

#### ﻿ Platydracus marmorellus

Taxon classificationAnimaliaColeopteraStaphylinidae

Fauvel, 1895

401AF809-64F3-5E4E-A5A6-992CD7347F52

Figs 3 , 5–8

Staphylinus marmorellus Fauvel, 1895: 253.
Platydracus marmorellus ; Herman 2001: 14.

##### Material examined.

China – **Hainan Prov.** • 1♀; Ledong County, Jianfengling N. R.; alt. 1000 m; 10–23 May 2011; BI Wen Juan leg.; SHNU • 2♀♀; Ledong County, Jianfengling, Mingfenggu; 18°44'N, 108°50'E; alt. 950 m; 30 Apr. 2012; Peng & Dai leg.; SHNU • 1♂1♀; Ledong County, Jianfengling N. R., Mingfenggu; 18°44'30″N, 108°50'29″E; rainforest, decaying log; alt. 995 m; 26 Jan. 2015; Peng, Yin, Tu, Song, Shen, Zhou & Wang leg.; SHNU • 1♀; Ledong County, Jianfengling N. R., District V; 18°43'57″N, 108°52'41″E; broad-leaved forest, decaying log; alt. 975 m; 24 Jan. 2015; Peng, Yin, Tu, Song, Shen, Zhou & Wang leg.; SHNU • 3♀♀; Qiongzhong County, Limu Mt., path to peak; 19°10'27″N, 109°45'29″E; mixed forest, decaying log; alt. 1000 m; 1 Feb. 2015; Peng, Tu, Song, Shen, Zhou & Yan leg; SHNU • 1♂1♀; Lingshui County, Diaoluoshan N. R.; 18°42'2.66″N, 109°53'19.32″E; alt. 950–1050 m; Aug. 2023; FIT; Zhao leg.; SHNU • 1♀; Ledong County, Jianfengling, Mingfenggu; 18°44'N, 108°50'E; alt. 950 m; 29 Apr. 2012; Peng & Dai leg.; SHNU.

**Figures 1–4.**
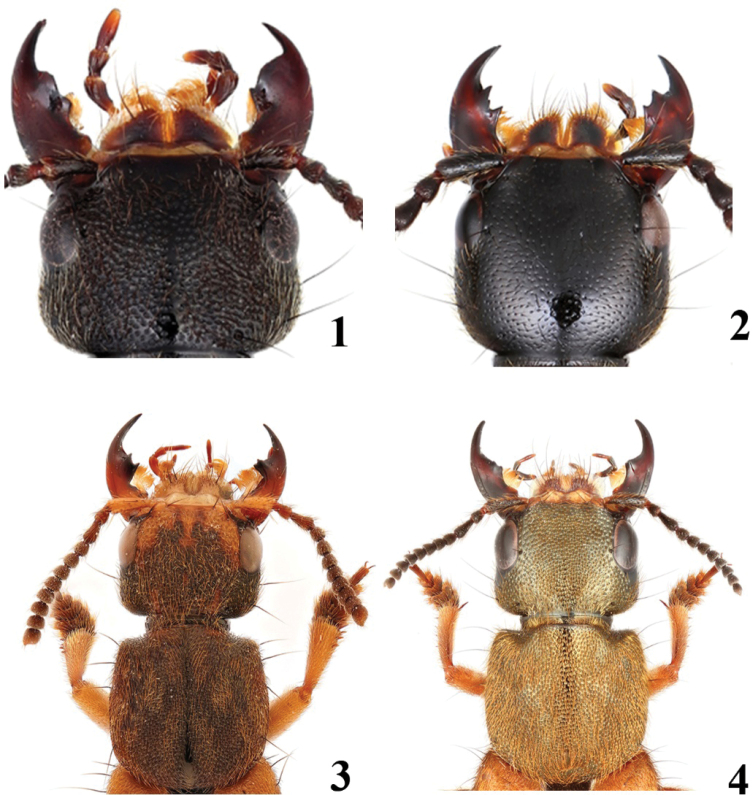
Punctation of head and setae on the head and pronotum **1***P.hainanensis***2***P.juang***3***P.marmorellus* with two types of setae **4***P.aureolus* with one type of setae.

##### Measurements.

***Male***: BL: 14.8–18.4 mm, FL: 8.78–9.22 mm. HL: 1.85–2.07 mm, HW: 2.59–2.82 mm, EYL: 0.82–0.89 mm, TL: 0.82–0.89 mm, PL: 2.90–3.21 mm, PW: 2.84–3.15 mm, EL: 3.21–3.89 mm, EW: 3.39–4.32 mm. HW/HL: 1.35–1.44, TL/EYL: 1.00, PL/PW: 1.02–1.13, EL/EW: 0.90–0.97. ***Female***: BL: 12.2–14.9 mm, FL: 7.67–9.11 mm. HL: 1.70–2.07 mm, HW: 2.15–2.45 mm, EYL: 0.82 mm, TL: 0.67–0.82 mm, PL: 2.53–3.02 mm, PW: 2.47–2.96 mm, EL: 2.96–3.39 mm, EW: 3.09–3.64 mm. HW/HL: 1.18–1.32, TL/EYL: 0.82–1.00, PL/PW: 1.00–1.03, EL/EW: 0.93–0.98.

**Figures 5–12.**
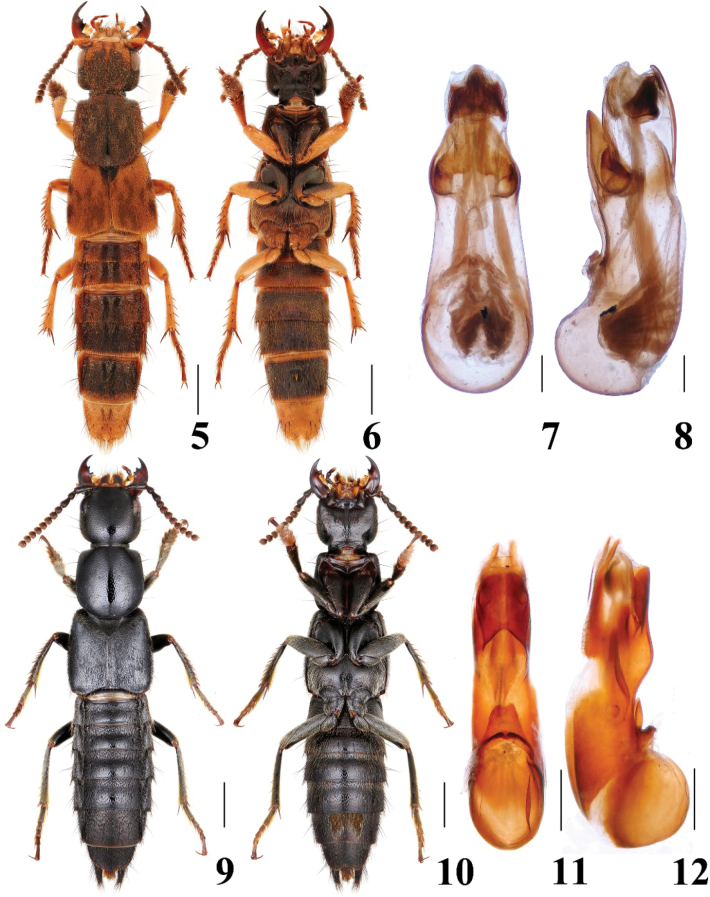
Habitus and aedeagus of *Platydracus***5–8***P.marmorellus***9–12***P.juang***5, 9** dorsal habitus **6, 10** ventral habitus **7, 11** aedeagus in ventral view **8, 12** aedeagus in lateral view. Scale bars: 2.0 mm (**5, 6**), 1.0 mm (**9, 10**), 0.2 mm (**7, 8**), 0.5 mm (**11, 12**).

##### Diagnosis.

The species is widely distributed in South China, and its detailed distributional information will be listed in a forthcoming paper by Qing-Hao Zhao. In general appearance, the species is similar to *P.plagiicollis* (Fairmaire, 1891) which overlaps in distribution. Yet, *P.marmorellus* can be easily distinguished from the latter by bearing pale appressed microsetae on the forebody, which are absent in *P.plagiicollis*.

##### Distribution.

China (Hainan), Myanmar, Vietnam, Indonesia. New to China.

#### ﻿ Platydracus juang

Taxon classificationAnimaliaColeopteraStaphylinidae

Smetana, 2005

FAC13E82-7D90-56DC-9731-7C1FE4DC42C7

Figs 2 , 9–12

Platydracus juang Smetana, 2005: 22; Xu and Tang 2019: 185; Hu 2020: 346; Zhou Zhao and Tang 2024: 508.

##### Material examined.

China – **Hainan Prov.** •1♂; Jianfengling N. R., decaying log; 15 Nov. 2009; Wen Dong leg.; SHNU • 1♀; Diaoluoshan N. R.; alt. 1000 m; Dec. 2022; Zhou & Cai leg.; SHNU • 1♀; Ledong County, Jianfengling N. R., Mingfenggu, rainforest, decaying log; 18°44'30″N, 108°50'29″E; alt. 995 m; 26 Jan. 2015; Peng, Yin, Tu, Song, Shen, Zhou & Wang leg.; SHNU • 1♀; Lingshui County; Diaoluo Shan N. R.; alt. 900 m; 22 May 2014; Qiu-Jian Yue leg.; SHNU.

##### Measurements.

***Male***: BL: 13.4–19.3 mm, FL: 6.89–10.55 mm. HL: 1.60–2.65 mm, HW: 1.91–2.84 mm, EYL: 0.62–0.80 mm, TL: 0.74–1.23 mm, PL: 2.28–3.39 mm, PW: 2.10–3.15 mm, EL: 2.71–4.07 mm, EW: 2.59–3.95 mm. HW/HL: 1.07–1.19, TL/EYL: 1.20–1.54, PL/PW: 1.08–1.15, EL/EW: 1.03–1.07. ***Female***: BL: 12.3–20.0 mm, FL: 7.00–9.67 mm. HL: 1.60–2.16 mm, HW: 1.97–2.59 mm, EYL: 0.56–0.74 mm, TL: 0.74–1.05 mm, PL: 2.34–3.15 mm, PW: 2.10–2.78 mm, EL: 2.71–3.70 mm, EW: 2.53–3.58 mm. HW/HL: 1.17–1.23, TL/EYL: 1.33–1.42, PL/PW: 1.11–1.13, EL/EW: 1.03–1.07.

##### Diagnosis.

The species is similar to *P.brachycerus* Smetana & Davies, 2000, in most aspects, but it can be distinguished from the latter by the more rounded temples, longer pronotum, and sparser punctation of the head.

##### Distribution.

China (Anhui, Fujian, Guangdong, Guangxi, Hainan, Hunan, Jiangxi, Taiwan, Zhejiang).

#### ﻿ Platydracus hainanensis sp. nov.

Taxon classificationAnimaliaColeopteraStaphylinidae

27A25ACC-1427-5BA7-92E8-F8FC04E8085E

https://zoobank.org/F1098124-7BC1-42FB-A2BF-48C5137DE7EC

Figs 1 , 13–16

##### Material examined.

***Holotype*.** China – **Hainan Prov.** • ♂; glued on a card with two labels as follows: “China: Hainan, Lingshui County, Diaoluoshan N. R., forest nr., residence; 18°44'N, 109°52'E; rainforest, decaying log; alt. 1100 m; 4 Feb. 2015; Peng, Yin, Tu, Song, Shen, Zhou, Yan & Wang leg.” “Holotype / *Platydracushainanensis*/ Huang, Chen & Tang” [red handwritten label]; SHNU. ***Paratypes*.** China – **Hainan Prov.** • 2♂♂; Lingshui County, Diaoluoshan N. R., forest nr., residence; 18°44'N, 109°52'E; rainforest, decaying log; alt. 1100 m; 4 Feb. 2015; Peng, Yin, Tu, Song, Shen, Zhou, Yan & Wang leg.; SHNU.

**Figures 13–20.**
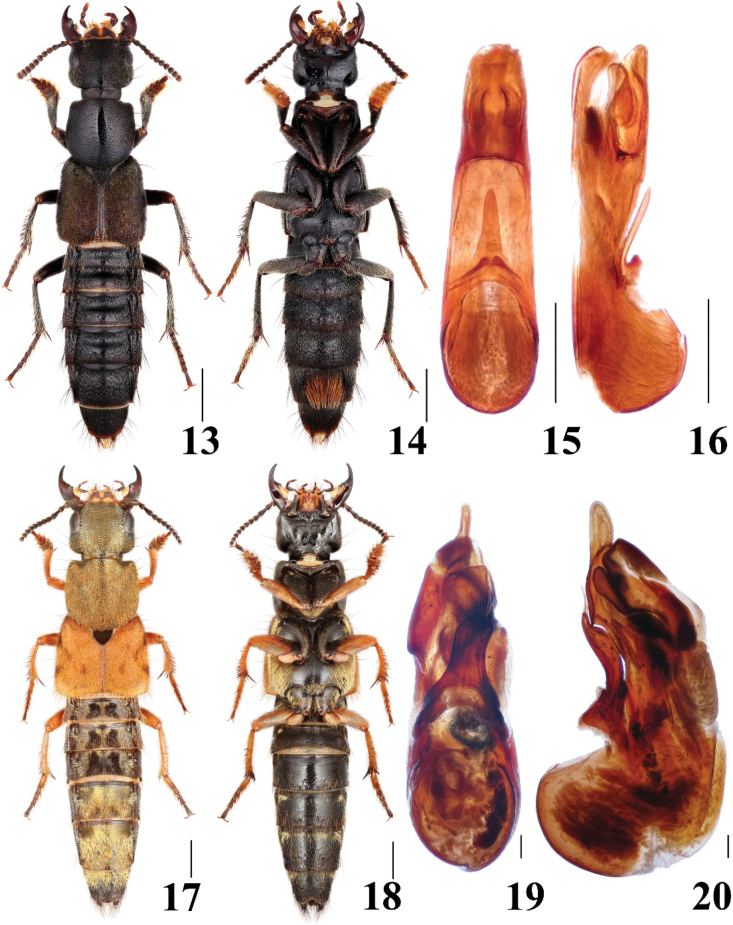
Habitus and aedeagus of *Platydracus***13–16***P.hainanensis***17–20***P.aureolus***13, 17** dorsal habitus **14, 18** ventral habitus **15, 19** aedeagus in ventral view **16, 20** aedeagus in lateral view. Scale bars: 1.0 mm (**13, 14**), 2.0 mm (**17, 18**), 0.5 mm (**15, 16**), 0.2 mm (**19, 20**).

##### Diagnosis.

The new species belongs to the *Platydracusbrachycerus* group, and it is similar to *P.smetanai* Zhou, Zhao & Tang, 2024 by sharing dense punctation of the head and short protarsomere 5. Yet, it can be distinguished from the latter by the distinctly larger body size (11.6–13.7 mm in *P.smetanai*) and a different aedeagus.

##### Description.

Measurements of male: BL: 15.3–17.8 mm, FL: 8.00–8.11 mm. HL: 1.67–1.85 mm, HW: 2.28–2.41 mm, EYL: 0.68 mm, TL: 0.80–0.86 mm, PL: 2.47–2.65 mm, PW: 2.41–2.59 mm, EL: 3.15–3.27 mm, EW: 3.21–3.33 mm. HW/HL: 1.30–1.37, TL/EYL: 1.18–1.27, PL/PW: 1.00–1.03, EL/EW: 0.98–1.02.

Body black, elytra with distinct copper tint, abdominal segments partially brownish, maxillary palpi, labial palpi and tarsomeres brownish with apical margin of each segment lighter, antennae dark brown with antennomeres gradually lighter apicad.

Head 1.30–1.37 times as wide as long; eyes moderately large, tempora 1.18–1.27 times as long as eye; surface with punctation dense and coarse; anterior portion of frons impunctate; pubescence most brownish except that behind anterior margin and eyes silver; antennomeres 1–3 distinctly longer than wide, antennomere 4 as long as wide, antennomeres 5–10 distinctly transverse, gradually incrassate, segments 4 to 11 covered with dense pubescence; maxillary palpi stout with last segment narrowly subtruncate apically; mandibles robust, inner edge of left mandible with two teeth on upper plane and mid tooth on lower plane, inner edge of right mandible with blunt anterior tooth and sharp posterior tooth on upper plane and mid tooth on lower plane.

Pronotum 1.00–1.03 times as long as wide; disc with narrow impunctate midline, punctation distinctly sparser than that of head, pubescence brownish; scutellum with dense rugulose punctation and dark pubescence.

Elytra 0.98–1.02 times as long as wide, punctation dense and coarse, pubescence behind humeri, along suture and posterior elytral margin silver, pubescence of the rest parts brownish.

Anterior legs with tibiae and tarsomeres distinctly stouter than those of the related species, tarsomere 5 rather short.

Abdominal tergites III–VI with basal transverse depression, with punctures coarser at base, gradually become finer apicad; pubescence brownish except for that of lateral portion of tergite III which is silver.

**Male.** Abdominal sternite VII with large median depression bearing numerous long dark brown setae; sternite VIII with posterior margin shallowly emarginate in the middle; aedeagus (Figs 15, 16) almost symmetrical, median lobe elongated with lateral sides subparallel forming broad round tip in ventral view; paramere elongated triangular, slightly curved ventrad in lateral view, without apical setae.

##### Etymology.

The specific epithet refers to the type locality of the new species.

##### Distribution.

China (Hainan).

#### ﻿ Platydracus aureolus sp. nov.

Taxon classificationAnimaliaColeopteraStaphylinidae

02973EF4-6CA4-58E6-B793-72E998D911B4

https://zoobank.org/32D8F009-3739-4806-B50A-BED52129DF57

Figs 4 , 17–20

##### Material examined.

***Holotype*.** China – **Hainan Prov.** • ♂; glued on a card with two labels as follows: “China: Hainan, Lingshui County, Diaoluoshan N. R.; 18°42'2.66″N, 109°53'19.32″E; alt. 950 m –1050 m; Aug. 2023; FIT; Zhao leg.” “Holotype / *Platydracusaureolus* / Huang, Chen & Tang” [red handwritten label]; SHNU. ***Paratypes*.** China – **Hainan Prov.** • 1♂; Lingshui County, Diaoluoshan N. R.; 18°42'2.66″N, 109°53'19.32″E; alt. 950 m–1050 m; Aug. 2023; FIT; Zhao leg.; SHNU • 7♂♂2♀♀; Lingshui County, Diaoluoshan N. R.; 18°42'2.66″N, 109°53'19.32″E, alt. 700 m; 9–16 Nov. 2022; FIT, Cai & Zhou leg; SHNU • 15♂♂6♀♀; Lingshui County, Diaoluoshan N. R.; 18°42'2.66″N, 109°53'19.32″E; alt. 950–1050 m; Aug. 2023; FIT; Zhao leg.; SHNU, NHMD. VIETNAM – **Vinh Phu Prov.** • 1♂; Tam Dao; 20–28 Jun. 1990; Mir. Dvořák leg.; NMPC • 1♂; Hanoi TAM DAO nat. park; 75 km; 15 May – 16 Jun. 1991; leg. [collector illegible]; CNC.

##### Diagnosis.

The new species can be easily recognized from other Chinese species by larger body size and distinct coloration.

##### Description.

Measurements of male: BL: 21.9–25.2 mm, FL: 9.33–10.78 mm. HL: 2.34–2.78 mm, HW: 3.09–3.58 mm, EYL: 1.18–1.32 mm, TL: 0.88–1.03 mm, PL: 3.52–4.20 mm, PW: 3.70–4.20 mm, EL: 4.07–5.12 mm, EW: 4.81–5.68 mm. HW/HL: 1.29–1.32, TL/EYL: 0.75–0.78, PL/PW: 0.95–1.00, EL/EW: 0.85–0.90.

Body mostly black, except head and pronotum with strong golden tint on dorsal side, elytra light reddish-brown with several small dark marks, maxillary palpi, labial palpi and mandibles partially reddish-brown, legs reddish-brown, except for the trochanters, basal profemora and basal mesofemora blackish, tibial spurs dark brown, tarsomeres especially terminal segment more or less dark.

Head 1.29–1.32 times as wide as long, eyes large, tempora 0.75–0.78 times as long as eye; surface with punctation dense and umbilicate; pubescence mostly golden; antennomeres 1–3 distinctly longer than wide, antennomere 4 as long as wide, antennomeres 5–10 distinctly transverse, gradually incrassate, segments 4 to 11 covered with dense pubescence; maxillary palpi slender; mandibles relatively slender, inner edge of left mandible with two small teeth and two teeth on lower plane, inner edge of right mandible with small tooth on upper plane and small tooth on lower plane.

Pronotum 0.95–1.00 times as long as wide; punctation similar to that of head, pubescence golden, relatively longer and more distinct than that of head; disc with small middle specular patch near posterior margin; scutellum with dense rugulose punctation and black pubescence.

Elytra 0.85–0.90 times as long as wide, punctation fine and dense, pubescence mostly golden. Mesoventrite and metaventrite covered with golden pubescence laterally.

Abdomen with tergites densely punctate; tergites III–V each with a pair of large dark tomentose spots, the rest portions covered with golden pubescence, tergite VI and VII each with a pair of small dark tomentose spots near posterior margin which can be indistinct in some specimens, the rest portions covered with golden pubescence though that of basolateral portions more or less dark, tergite VIII covered with golden pubescence on basal two-thirds, and tergite VII with apical palisade fringe.

**Male.** Abdominal sternite VIII with posterior margin emarginate in the middle; aedeagus (Figs 19, 20) asymmetrical, twisted to the right side in ventral view; median lobe with slender and elongate tip; paramere broad, with apical setae, subapical portion with cavity to accommodate the large apical tooth of the median lobe.

##### Etymology.

The specific epithet refers to the coloration of the new species.

##### Distribution.

China (Hainan).

#### ﻿ Platydracus zhouchenglini sp. nov.

Taxon classificationAnimaliaColeopteraStaphylinidae

B8A1ECDE-A421-5961-A587-B15CB3ADCCE4

https://zoobank.org/B4A1550D-C5D6-4B35-8E93-8A9C2AD749E5

Figs 21–24

##### Material examined.

***Holotype*.** China – **Hainan Prov.** • ♂; glued on a card with two labels as follows: “China: Hainan, Lingshui County, Diaoluoshan N. R.; 18°42'2.66″N, 109°53'19.32″E; alt. 700 m; 9–16 Nov. 2022; rotten meal trap; Cai & Zhou leg.” “Holotype / *Platydracuszhouchenglini* / Huang, Chen & Tang” [red handwritten label]; SHNU. ***Paratypes*.** China – **Hainan Prov.** • 2♂♂1♀; Lingshui County, Diaoluoshan N. R.; 18°42'2.66"N, 109°53'19.32"E; alt. 950–1050 m; Aug. 2023; FIT; Zhao leg.; SHNU • 2♂♂; Lingshui County, Diaoluoshan N. R.; 18°42'2.66"N, 109°53'19.32"E; alt. 700 m; 9–16 Nov. 2022; rotten meat trap; Cai & Zhou leg.; SHNU • 1♀; Lingshui County, Diaoluoshan N. R., Houshan; 18°43'46.81"N, 109°51'38.52"E; alt. 900–1250 m; 13–14 Nov. 2022; Cai & Zhou leg.; SHNU • 1♀; Lingshui County, Diaoluoshan N. R., Houshan; 18°43'46.81"N, 109°51'38.52"E; alt. 900–1250 m; 11–12 Nov. 2022; Cai & Zhou leg.; SHNU • 3♂♂7♀♀; Ledong County, Jianfengling, Mingfenggu; 18°44'N, 108°50'E; alt. 950 m; 30 Apr. 2012; Peng & Dai leg.; SHNU • 1♂; Wuzhishan Mt., Guanshandian; 18°53'N, 109°41'E; alt. 650 m; 20 Apr. 2012; Yin Ziwei leg.; SHNU • 1♂; Lingshui County, Diaoluoshan Mt., Winding Road; 18°42'N, 109°52'E; alt. 600–1000 m; 26 Apr. 2012; Peng & Dai leg.; SHNU • 1♂; Wuzhishan Mt., Guanshandian; 18°53'N, 109°41'E; alt. 700 m; 21 Apr. 2012; Peng & Dai leg.; SHNU • 1♀; Ledong County, Jianfengling N. R., Mingfengfu; 18°44'30"N, 108°50'29"E; rainforest, decaying log; alt. 995 m; 28 Jan. 2015; Peng, Yin, Tu, Song, Shen, Zhou & Wang leg.; SHNU • 1♀; Changjiang County, Bawangling N. R.; 11 Apr. 2010; alt. 1000 m; YIN Z. W. leg.; SHNU • 5♂♂6♀♀; Lingshui County, Diaoluoshan N. R.; 18°42'2.66"N, 109°53'19.32"E, alt. 700 m; 9–16 Nov. 2022; FIT, Cai & Zhou leg; SHNU • 65♂♂36♀♀; Lingshui County, Diaoluoshan N. R.; 18°42'2.66"N, 109°53'19.32"E; alt. 950–1050 m; Aug. 2023; FIT; Zhao leg; SHNU, NHMD.

##### Diagnosis.

**Figures 21–28.**
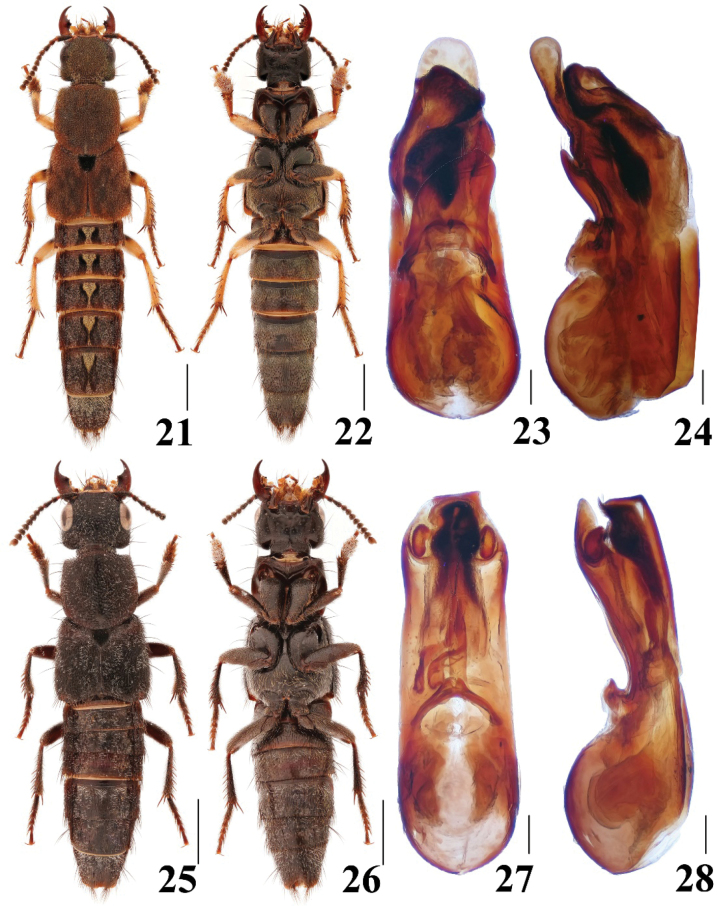
Habitus and aedeagus of *Platydracus***21–24***P.zhouchenglini***25–28***P.subirideus***21, 25** dorsal habitus **22, 26** ventral habitus **23, 27** aedeagus in ventral view **24, 28** aedeagus in lateral view. Scale bars: 2.0 mm (**21, 22, 25, 26**), 0.2 mm (**23, 24, 27, 28**).

The new species can be easily recognized from other Chinese species by abdominal tergites III–VII with median golden tomentose patches that are continuous along the midline (Fig. 21).

##### Description.

Measurements of male: BL: 9.6–16.8 mm, FL: 6.67–9.22 mm. HL: 1.42–1.85 mm, HW: 1.91–2.53 mm, EYL: 0.74–0.93 mm, TL: 0.37–0.74 mm, PL: 2.41–3.27 mm, PW: 2.34–3.02 mm, EL: 2.84–3.70 mm, EW: 2.84–3.76 mm. HW/HL: 1.24–1.39, TL/EYL: 0.40–0.80, PL/PW: 1.03–1.08, EL/EW: 0.94–1.02.

Measurements of female: BL: 11.7–16.7 mm, FL: 6.78–9.00 mm. HL: 1.48–1.79 mm, HW: 1.91–2.41 mm, EYL: 0.93 mm, TL: 0.37–0.62 mm, PL: 2.41–3.15 mm, PW: 2.28–2.96 mm, EL: 2.78–3.52 mm, EW: 2.90–3.64 mm. HW/HL: 1.29–1.40, TL/EYL: 0.40–0.67, PL/PW: 0.95–1.06, EL/EW: 0.94–0.97.

Body dark brown, pronotum reddish-brown along the margins, elytra reddish-brown with dark marks, maxillary palpi, labial palpi and mandibles reddish-brown, legs reddish-yellow, except for the trochanters and base of femora blackish, tibial spurs dark brown, tarsomeres more or less dark.

Head 1.24–1.40 times as wide as long, eyes large, tempora 0.40–0.80 times as long as eye; surface with punctation dense and umbilicate; pubescence mostly brownish; antennomeres 1–3 distinctly longer than wide, antennomere 4 slightly shorter than wide, antennomeres 5–10 distinctly transverse, gradually incrassate, segments 4 to 11 covered with dense pubescence; maxillary palpi slender; mandibles relatively slender, inner edge of left mandible with two acute teeth and two teeth on lower plane, inner edge of right mandible with round tooth and acute tooth on upper plane and acute tooth on lower plane.

Pronotum 1.03–1.08 times as long as wide; punctation and pubescence similar to that of head; disc with narrow impunctate midline in posterior one-fifth; scutellum with dense rugulose punctation and black pubescence.

Elytra 0.94–1.02 times as long as wide, punctation fine and dense, pubescence comprised of two types of setae, long setae mostly brownish, short setae mostly whitish.

Abdomen with tergites densely punctate; tergites III–VII each with median golden tomentose patch and a pair of large dark tomentose spots, the lateral portions covered with long brown setae and short white setae, tergite VIII covered with golden pubescence which is distinctly sparse in apical third, tergite VII with apical palisade fringe.

**Male.** Abdominal sternite VIII with posterior margin emarginate in the middle; aedeagus (Figs 23, 24) asymmetrical, twisted to right side in ventral view; median lobe with broad and round tip; paramere broad, with apical setae, subapical portion with cavity to accommodate apical tooth of the median lobe.

##### Etymology.

This species is named in honor of Mr Cheng-Lin Zhou who collected many *Platydracus* specimens.

##### Distribution.

China (Hainan).

#### ﻿ Platydracus subirideus

Taxon classificationAnimaliaColeopteraStaphylinidae

Kraatz, 1859

F4A18245-1CA6-56E0-83E6-3F6BBA9E5431

Figs 25–28

Staphylinus subirideus Kraatz, 1859: 78; Cameron 1932: 199.
Platydracus subirideus ; Herman 2001: 14.

##### Material examined.

China – **Hainan Prov.** • 1♂; Lingshui County, Mt. Diaoluoshan Reservoir; 18°43'N, 109°53'E; alt. 950–1000 m; 25 Apr. 2012; PAN Y. H. & LI W. R. leg.; SHNU.

##### Measurements.

Male: BL: 12.3 mm, FL: 6.66 mm. HL: 1.74 mm, HW: 2.19 mm, EYL: 0.86 mm, TL: 0.56 mm, PL: 2.37 mm, PW: 2.36 mm, EL: 2.58 mm, EW: 2.88 mm. HW/HL: 1.26, TL/EYL: 0.65, PL/PW: 1.00, EL/EW: 0.90.

##### Diagnosis.

The species is similar to *P.maculipennis* (Kraatz, 1859) in most aspects, but it can be recognized from the latter by the posterolateral pale portion of elytra small and indistinctly yellowish (large and distinctly yellowish in *P.maculipennis*), abdomen with whitish setae (yellowish in *P.maculipennis*) and legs blackish (yellowish in *P.maculipennis*).

##### Distribution.

China (Hainan). Described from ‘East Indies’. New to China.

## Supplementary Material

XML Treatment for Platydracus marmorellus

XML Treatment for Platydracus juang

XML Treatment for Platydracus hainanensis

XML Treatment for Platydracus aureolus

XML Treatment for Platydracus zhouchenglini

XML Treatment for Platydracus subirideus

## ﻿Additional information

### Author ORCIDs

Jia-Yi Huang https://orcid.org/0009-0005-4612-5313

Jia-Wei Chen https://orcid.org/0009-0001-4757-9406

Liang Tang https://orcid.org/0000-0002-6731-4544

### Data availability

All of the data that support the findings of this study are available in the main text.
